# Functional distinctions in cytosolic calcium regulation between cells of the glomerular filtration barrier

**DOI:** 10.1016/j.ceca.2010.06.005

**Published:** 2010-07

**Authors:** Rebecca Rachael Foster, Gavin I. Welsh, Simon C. Satchell, Robin D. Marlow, Mathew D. Wherlock, Debora Pons, Peter W. Mathieson, David O. Bates, Moin A. Saleem

**Affiliations:** a The Academic Renal Unit, Southmead Hospital, University of Bristol, BS10 5NB, UK; b Microvascular Research Laboratories, Department of Physiology, University of Bristol, BS2 8EJ, UK

**Keywords:** Flufenamic acid, TRPC6, Glomerular endothelial cells, Podocytes, Calcium

## Abstract

The importance of intracellular calcium ([Ca^2+^]_i_) regulation in the glomerular filtration barrier (GFB) has recently been highlighted by mutations in the cation channel TRPC6, resulting in a renal-specific phenotype. We examined the effects of FFA, a tool that can activate TRPC6, on [Ca^2+^]_i_ in human conditionally immortalised glomerular endothelial cells (ciGEnC) and human podocytes (ciPod) that form the GFB. Changes in [Ca^2+^]_i_ stimulated by FFA were measured in Fura 2-AM loaded cells. In GEnC, cell activation by FFA was dependent on external Ca^2+^, yet in ciPod it was not. Depletion of internal Ca^2+^ stores with thapsigargin did not affect cell activation by FFA in ciGEnC, but inhibited it in ciPod in a nephrin-dependent manner, demonstrated using nephrin deficient (ND) ciPod in conjunction with nephrin rescue experiments. FFA induced [Ca^2+^]_i_ store release in ciPod, but not in ciGEnC or ND ciPod. In parallel, there were differences in the localisation of overexpressed TRPC6 between ciGEnC and ciPod. Furthermore, co-transfection of nephrin with TRPC6 in HEK293 cells reduced the FFA-induced increase in [Ca^2+^]_i_ and nephrin clustering altered TRPC6 distribution. In conclusion, cell activation by FFA in podocytes stimulates the opening of a Ca^2+^ channel, probably TRPC6, in a nephrin-dependent manner with a different activation profile to GEnC.

## 1 Introduction

Flufenamic acid is a non-steroidal anti-inflammatory agent belonging to the family of fenamates. It has been used historically to block Ca^2+^ influx through canonical transient receptor potential channels (TRPC), a family of non-selective cation channels, and has other channel properties such as inducing the release of Ca^2+^ from mitochondria [1], potentiating large conductance *K*_Ca_ channels and inhibiting Ca^2+^ activated Cl^−^ channels and L-type voltage gated Ca^2+^ [2]. In 2001, Inoue et al. [3] demonstrated that FFA could activate TRPC6, whilst inhibiting other TRPCs. Since then a number of groups have confirmed this effect [4–9]. Native TRPC6 Ca^2+^ activation is particularly difficult to isolate, therefore this observation was promising, especially since TRPC6 plays such an important role in many systems throughout the body including nervous systems [10,11], blood [12,13] and smooth muscle and endothelial cells of the cardiovascular [6,14] and pulmonary systems [15]. TRPCs are activated downstream of phospholipase-C (PLC) [16] and are defined by whether they are directly activated by DAG, termed receptor operated channels (TRPC3, TRPC6 and TRPC7) [17], or whether they are activated by inositol 1,4,5-tris-phosphate (IP_3_) induced depletion of cell Ca^2+^ stores, such as the endoplasmic reticulum, termed store operated channels (SOC) (TRPC1, TRPC4 and TRPC5) [18,19].

In 2005, a missense mutation in exon 2 of the *TRPC6* gene was identified in a large family with a high incidence of late onset autosomal dominant hereditary focal and segmental glomerulosclerosis (FSGS), a renal specific pathology [20]. All individuals affected shared the same mutation. Reiser et al. [21] demonstrated a further five families with inherited FSGS who presented with heterozygous sequence changes in *TRPC6*. Overall 3 of the 6 described hereditary TRPC6 mutations caused increased Ca^2+^ influx when transfected into HEK293 cells [20,21], but importantly pathological effects were only observed in the kidneys despite widespread expression throughout the body. The glomerular filtration barrier, which consists of highly fenestrated glomerular endothelial cells, a glomerular basement membrane and glomerular epithelial cells or podocytes is highly disrupted in FSGS. Podocyte foot processes interdigitate with each other forming the ‘slit diaphragm’ which consists of a complex of specialised proteins, many of which are restricted to podocytes such as the cell adhesion molecule nephrin [22].

Intracellular Ca^2+^ is a secondary messenger commonly involved in a number of cellular signalling pathways, such as differentiation, apoptosis, proliferation and cell contraction. Recently, the focus on Ca^2+^ signalling in podocytes has been directed to TRPC6 signalling, which is thought to act as a mechanosensor. Entry of Ca^2+^ through TRPC6 modulates the actin cytoskeleton resulting in foot process “effacement”, leading to loss of size selectivity (as reviewed in [23]). The role of Ca^2+^, and in particular TRPC6, signalling in glomerular endothelial cells has not been explored to the same extent. TRPC6 however has been studied in microvascular endothelial cells in other systems throughout the body, and shown to be involved in vascular tone and vascular permeability [24]. Therefore TRPC6 Ca^2+^ (mis)signalling has important implications for both cell types of the GBF in the progression of FSGS.

We have recently further explored the Ca^2+^ enhancing properties of FFA on TRPC6, specifically in cultured podocytes [25], demonstrating that FFA increased cytosolic Ca^2+^ in a TRPC6 dependent manner, in contrast to other fenamates. Furthermore TRPC3 and TRPC7, which can form heterotetramers with TRPC6 did not enhance FFA induced Ca^2+^ activation. Since both endothelial cells and podocytes form the filtration barrier and both potentially use TRPC6 to regulate Ca^2+^ entry, we went on to compare and contrast FFA Ca^2+^ activation within podocytes and glomerular endothelial cells, in addition to TRPC6 protein analysis. Our results suggest that the pattern of FFA induced cytosolic Ca^2+^ influx in podocytes is distinct from GEnC and other described cell types, and is at least partially controlled by the podocyte specific protein nephrin.

## 2 Experimental procedures

### 2.1 Chemicals

All chemicals and solutions and antibodies were obtained from Sigma Chemical Co. (St Louis, MO) unless otherwise stated.

### 2.2 Antibodies

A rabbit polyclonal human anti-TRPC6 antibody (Alomone, Jerusalem, Israel) was used for immunofluorescence and Western blotting. A mouse monoclonal human anti β-actin antibody was also used for Western blotting.

### 2.3 Cell culture

Normal human podocytes were conditionally immortalised with a temperature sensitive mutant of SV-40 large T-antigen (ciPod). These cells have been previously characterised in detail elsewhere [26]. At the “permissive” temperature of 33 °C the transgene is active, and allows the cells to proliferate rapidly. Thermoswitching the cells to the “non-permissive” temperature of 37 °C inactivates the transgene and the cells become growth arrested and differentiate to express antigens appropriate to *in vivo* arborised podocytes. To assess the significance of nephrin in the functional responses investigated another cell line was studied. This was a ciPod cell line isolated from a patient with congenital Finish nephrotic syndrome (Fin_major_)—nephrin deficient (ND) ciPod [27]. All podocytes were grown in 10% RPMI media containing 1% penicillin/streptomycin (Gibco-Invitrogen, Carlsbad, CA) and 1× ITS liquid media.

Normal human GEnC were also conditionally transformed (ciGEnC), using the same technique as that described above. These cells have been previously characterised in detail elsewhere [28] and can also be thermoswitched to inactivate the SV-40 large T antigen transgene. These cells were grown in endothelial growth medium-2 (EGM-2 MV, Cambrex, Wokingham, UK) containing 5% foetal calf serum and supplements as supplied, excepting VEGF. HEK 293 cells were also maintained in 10% RPMI media containing 1% penicillin/streptomycin.

### 2.4 Western blotting

Differentiated ciPod, and ciGEnC, platelets and glomeruli were lysed on ice in Triton X-100 lysis buffer (20 mM Tris pH 7.5, 1.5% Triton X-100, 150 mM NaCl, 10% glycerol, 1 mM EDTA) containing a proteinase inhibitor cocktail (1:100 dilution). The samples were cleared by centrifugation at 13,000 rpm for 3 min at 4 °C and the pellet discarded. Total protein was then quantified by bicinchoninic acid assay according to manufacturer's instructions (Pierce Chemical Co., Rockford, IL). Protein samples were separated by SDS-PAGE under reducing conditions and were transferred to nitrocellulose membranes. The membranes were blocked in 10% fat-free milk before incubation with antibodies described above. After incubation with horseradish peroxidase-conjugated secondary antibodies (Santa Cruz Biotechnology, Santa Cruz, CA), bands were detected using the SuperSignal West Femto Maximum Sensitivity Substrate (Pierce Chemical Co.) and imaged using the Chemidoc-IT system (UVP Bioimaging systems, Upland, CA).

### 2.5 Microinjection of differentiated ciPods and imaging

Full-length wild type (WT) TRPC6 cDNA incorporated in a pcDNA3 vector was a kind gift of Professor Thomas Gudermann (Marburg, Germany). Twenty to 100 μg/ml WT TRPC6 plasmid (in 0.4 mM Tris–HCl, 0.04 mM EDTA, pH 8.0) was microinjected into podocytes, using an Eppendorf AG semi-automatic microinjection system (Hamburg, Germany). After microinjection, the cells were incubated in RPMI media, containing supplements described previously, for 24 h prior to immunofluorescence. Cells were then fixed in 4% paraformaldehyde, permeabilised in 0.3% Triton X-100/PBS for 5 min and blocked in 5% FCS/0.05% Tween for 30 min. Cells were then incubated in 4 μg/ml primary anti-TRPC6 antibody for 1 h, washed and then incubated in 1:200 dilution Alexa Flour 488 conjugated anti-rabbit secondary antibody (Santa Cruz) for 30 min. Coverslips were washed and mounted using Vectashield mounting medium (Vector Laboratories, Burlingame, CA). Confocal microscopy was performed using a Leica SP2 confocal microscope (Leica Microsystems, Wetzlar, Germany).

### 2.6 Intracellular calcium ([Ca^2+^]_i_) measurements

Cells on 22 mm diameter glass coverslips were incubated with Fura 2-AM (10 μM) with 0.006% pluronic (Molecular Probes, Leiden, Netherlands) for ∼90 min in RPMI/EGM-2 containing serum at 37 °C. Changes in fluorescent intensity were analysed as described previously [29].

Experiments were conducted in Krebs-Ringer phosphate buffer (150 mM NaCl, 6 mM KCl, 1 mM MgCl_2_, 10 mM d-glucose and 10 mM HEPES) containing normal Ca^2+^ (1.5 mM CaCl_2_) or minimal CaCl_2_ (4 ± 0.4 μM, confirmed with Fura pentapotassium salt by reading against a full in vitro Ca^2+^ calibration curve), thereby reducing the external Ca^2+^ concentration gradient. We chose not to fully chelate Ca^2+^ because this would create a Ca^2+^ gradient out of the cell, which may affect Ca^2+^ kinetics. Where minimal Ca^2+^ was used, 10 μM ionomycin (IM) only induced a minimal increase in [Ca^2+^]_i_ following stimulation with 200 nM thapsigargin (TG, an inhibitor of sarco-endoplasmic Ca^2+^ ATPases, Calbiochem, San Diego, CA), demonstrating that the external Ca^2+^ concentration was successfully reduced (data not shown).

Cells were perfused with buffer (normal or minimal Ca^2+^) for 10 min. Cells were then perfused continuously with buffer containing stimulus or vehicle for 500 s. Each condition was applied to a separate coverslip to avoid the introduction of extraneous variables. Two hundred nanomolar TG was used to passively deplete stores. This concentration was chosen since it induced maximal store depletion in all cell lines (data not shown). To ensure that [Ca^2+^]_i_ could be measured in each cell population 10 μM IM was added, stimulating Ca^2+^ entry into the cells. 5 mM manganese chloride (MnCl_2_), in the continued presence of 10 μM IM was then used to quench the Ca^2+^ sensitive Fura to determine the (Ca^2+^-independent) background fluorescence signal. The normalised ratio (*R*_norm_), proportional to the Ca^2+^ concentration, was calculated as described previously [30]. To compare representative *R*_norm_ traces between experiments the *R*_norm_ was expressed as fold change from baseline for that experiment (change in Ca^2+^). Data are expressed as the area under the curve after 500 s (AUC_500_), calculated as shown below:

$$\text{AU}{\text{C}}_{500}{{=}_{500}}^{1}\sum (T_{n}-T_{\left(n-1\right)}).\left(\frac{\left(R_{n}+R_{n}\right)}{2}\right)$$

i.e. the sum (Σ) of the areas under the curve between the first (*n* = 1) and the 500th (*n* = 500) time point (*T*) at 0.25 s intervals after the start (*T* = 0) for the mean fold change in baseline *R*_norm_ (*R*) between each time point ((*R*_*n*_ + *R*_(*n*−1)_)/2). This approximates to the area under the curve when the average rate of change of the ratios is linear between time points [i.e. ((*R*_*n*_ + *R*_(*n*−1)_)/2) approximates to the average ratio between time points *n* and *n* − 1]. Where TG was used to deplete stores in the presence of normal external Ca^2+^, [Ca^2+^]_i_ levels were much higher due to capacitative Ca^2+^ entry, which can inhibit channel activity. Under these circumstances only the positive integers of the area under the curve were measured (+AUC_500_).

### 2.7 Transfection of cells

70% confluent proliferating ND ciPods or differentiated GEnC were grown on coverslips in 6 well plates and transfected with 2.5 μg pcDNA3.1 vector alone or containing full-length wild type nephrin in pcDNA3.1 (a kind gift of Harry Holtherfer, University of Helsinki, Finland) using Genejuice (Invitrogen) as instructed by the manufacturers. HEK293 cells were seeded on coverslips at 60–70% confluency and were transfected with 2.5 μg WT TRPC6 with 2.5 μg pcDNA3.1, 2.5 μg CD16-nephrin (wild type) containing a GFP tag (a kind gift Nina Jones, University of Guelph, Canada [31]) with 2.5 μg pcDNA3.1 or TRPC6 and CD16-nephrin using Genejuice. Transfection reagent was removed after 2 h and cells were maintained in normal media until used 24 h later.

### 2.8 Nephrin clustering and immunofluoresence

Transfected HEK293 cells were incubated with 1 μg/ml mouse anti-human CD16 (Santa Cruz) for 10 min at 37 °C or left untreated, then incubated with 1 μg/ml anti-mouse secondary conjugated to AF488 for 10 min at 37 °C. These time courses were chosen because CD16-nephrin is phosphorylated yet still remains predominantly at the cell surface [31]. Cells were then fixed in 4% paraformaldehyde, permeabilised in 0.3% Triton X-100/PBS for 10 min and blocked in 5% BSA/PBS for 30 min. Cells were then incubated in 8 μg/ml rabbit anti-human TRPC6 for 1 h, then incubated in 1 μg/ml anti-rabbit secondary conjugated to AF568 (Santa Cruz). Coverslips were mounted using Vectorshield mounting medium containing Dapi (Vector Laboratories). Cells were imaged using an AF600 LX fluorescence microscope (Leica Microsystems).

### 2.9 Nephrin clustering and [Ca^2+^]_i_ measurements

Transfected HEK293 cells, loaded with Fura-2 as above, were incubated with 1 μg/ml mouse anti-human CD16 in Krebs’ buffer for 10 min or left untreated, then incubated with 1 μg/ml normal mouse IgG (Santa Cruz) for 10 min in Krebs’ buffer. Cells were then stimulated with 200 μM FFA as described above.

### 2.10 Statistical analysis

Microsoft Office Excel (Microsoft Corporation, Redmond, Washington State) was used for simple statistics (paired and unpaired *t*-tests) and Prism (GraphPad, Oxford, UK) was used for ANOVA. Data are presented as mean ± S.E.M and *p* < 0.05 was taken to indicate statistical significance.

## 3 Results

### 3.1 The FFA induced Ca^2+^ signalling is dependent on external Ca^2+^ in ciGEnC, but not in ciPod

**Fig. 1:**
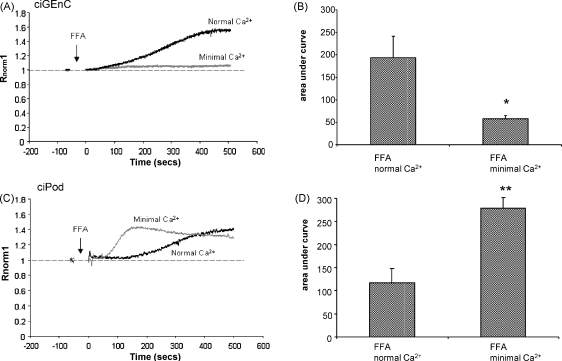
The FFA induced Ca^2+^ signalling is dependent on external Ca^2+^ in ciGEnC, but not in ciPod. ciGEnC and ciPod were loaded with Fura-2AM and stimulated with 200 μM FFA in either Krebs-Ringer phosphate buffer containing normal or minimal. (A) Representative traces of ciGEnC stimulated with FFA in normal and minimal extracellular Ca^2+^. (B) Summary of area under the curve for experiments in (A) (paired *t*-test, *n* = 9 and 6 respectively). (C) Representative trace of ciPod stimulated with FFA in normal and minimal extracellular Ca^2+^. (D) Summary of area under the curve for experiments in (C) (unpaired *t*-test, *n* = 7 and 6 respectively). *p < 0.05, **p < 0.01.

Changes in [Ca^2+^]_i_ (*R*_norm_) were used as a measure of channel activity. Firstly we confirmed that ciGEnC were activated by FFA. ciGEnC were incubated in Fura-2AM, and perfused with buffer containing either vehicle or FFA (Fig. 1A). FFA induced an increase in [Ca^2+^]_i_ in the presence of normal extracellular Ca^2+^ (AUC_500_; 194 ± 51 (Fig. 1B, compared with vehicle; −36.9 ± 27, *p* < 0.01 unpaired *t*-test, (data not shown)), confirming that FFA induces an increase in [Ca^2+^]_i_ in ciGEnC. This Ca^2+^ influx was reduced in limited extracellular Ca^2+^ (AUC_500_; 57 ± 8, *p* < 0.05 compared with normal Ca^2+^, unpaired *t*-test, Fig. 1A and B), demonstrating similarities with TRPC6 activation as described in other systems [17]. ciPod were treated in the same way as ciGEnC to compare and contrast cell activation by FFA. In ciPod perfused in buffer containing normal Ca^2+^, FFA induced an increase in [Ca^2+^]_i_ as expected and was not significantly different to that in ciGEnCs (*p* > 0.05, unpaired *t*-test). Yet in minimal Ca^2+^ the response was surprisingly significantly larger (AUC_500_ normal Ca^2+^; 117 ± 31 compared to minimal Ca^2+^; 393.8 ± 33 *p* < 0.01, unpaired *t*-test, Fig. 1C and D). This suggests that, if FFA is activating TRPC6 in ciPod, this response is not dependent on external Ca^2+^ in ciPod as suggested previously [25], and the effect of FFA on the [Ca^2+^]_i_ increase in ciPods is different from that in ciGEnC.

### 3.2 The FFA induced Ca^2+^ activation is store-independent in GEnC but is store-dependent in ciPod

**Fig. 2:**
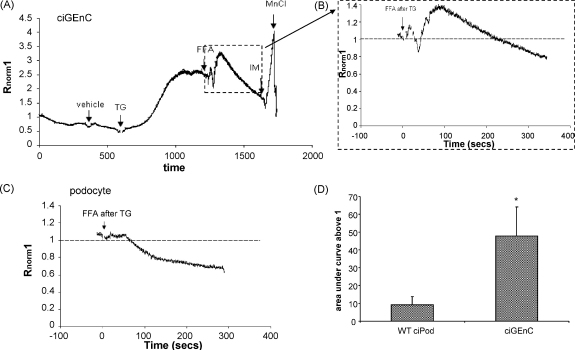
The FFA induced Ca^2+^ activation is store-independent in GEnC but is store-dependent in ciPod. GEnC and ciPods loaded with Fura-2AM were pre-incubated with TG to deplete stores, then stimulated with FFA. (A) An example of an entire experiment in ciGEnC showing response to vehicle, TG then FFA, ionomycin (IM) then quench (MnCl). (B) Representative trace of the response to FFA in GEnC after store depletion, taken from (A). (C) Representative trace of the response to FFA in ciPod after store depletion. (D) Summary of data represented in (B) and (C) presented as area under the curve above 1, unpaired *t*-test, *n* = 6 and 5 respectively *p < 0.05.

Typically, TRPCs either induce Ca^2+^ influx upon activation by DAG, or upon activation by depletion of Ca^2+^ from internal stores (store-dependent). To investigate whether it is possible that the differences seen in FFA induced Ca^2+^ activation involve the depletion of internal stores in either ciGEnC or ciPod, the effects of FFA were examined after the depletion of Ca^2+^ stores in normal external Ca^2+^. When stores were depleted using 200 nM thapsigargin (TG), FFA continued to induce an increase in [Ca^2+^]_i_ in ciGEnC (Fig. 2A and B) but not in ciPod (Fig. 2C) (+AUC_500_; ciGEnC; 47.8 ± 16.3 compared to ciPod; 9 ± 4.6, *p* < 0.05, unpaired *t*-test).

These results also suggest that the FFA-induced rises in [Ca^2+^]_i_ in ciGEnC are dependent on the entry of extracellular Ca^2+^ and not the release of Ca^2+^ from intracellular stores (as expected for the activation of TRPC6), whereas those in ciPods depend on the release of Ca^2+^ from intracellular stores.

### 3.3 The FFA induced Ca^2+^ activation is store-independent in the absence of nephrin in ciPods

**Fig. 3:**
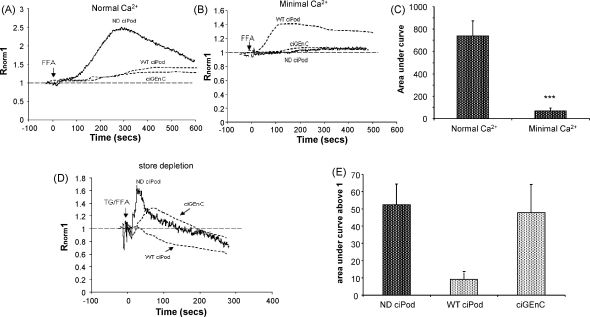
The FFA induced Ca^2+^ activation is store-independent in the absence of nephrin in ciPods. ND ciPods were loaded in Fura-2AM, then stimulated with FFA in normal or minimal extracellular Ca^2+^ with or with out pre-incubation with thapsigargin (TG). (A) Representative trace of ND ciPod stimulated with FFA in normal Ca^2+^ compared to previous results in WT ciPod and ciGEnC (dashed lines). (B) Representative trace of ND ciPod stimulated with FFA in minimal Ca^2+^ compared to previous results in WT ciPod and ciGEnC (dashed lines). (C) Summary of mean area under the curve for ND ciPod experiments in (A) and (B), *n* = 6 and 6. Unpaired *t*-test. (D) Representative trace demonstrating the effect of FFA after depletion of stores in ND ciPod compared to previous results in WT ciPod and ciGEnC (dashed lines). (E) Summary of area under the curve above 1 for experiments shown in (D), including previous results for WT ciPod and ciGEnC, One way ANOVA *n* = 5, *p* < 0.05. ***p < 0.001.

TRPC6 is associated with the slit diaphragm proteins nephrin and podocin and its expression is upregulated in renal biopsies of 2-day-old nephrin-deficient mice, forming aggregates reportedly in the podocytes [21]. Thus nephrin could be a potential candidate to affect typical TRPC6 activation in podocytes. To examine the functional effects of nephrin on cell activation by FFA, a nephrin deficient ciPod cell line (ND ciPod) was used. ND ciPod, loaded in Fura-2AM were perfused with buffer containing either normal or minimal Ca^2+^ and stimulated with FFA with or without pre-incubation with TG. We observed that the overall increase in [Ca^2+^]_i_ induced by FFA was much greater in ND ciPod than WT ciPod (dashed line, Fig. 3A, *p* < 0.05, unpaired *t*-test), suggesting that the absence of nephrin expression enhanced FFA induced signalling. In contrast to WT ciPod this increase in [Ca^2+^]_i_ was blocked by reducing external Ca^2+^ (AUC_500_: 68.7 ± 25.6 s compared to normal Ca^2+^: 740.11 ± 134.5 s *p* < 0.001 unpaired *t*-test, Fig. 3C). Also in contrast to WT ciPod, but similar to ciGEnC, FFA could still induce an increase in [Ca^2+^]_i_ in ND ciPods after the depletion of internal stores by TG (+AUC_500_; 52.3 ± 12, one way ANOVA *p* < 0.05 Fig. 3D and E). This demonstrates that in ciPod that do not express nephrin the FFA induced Ca^2+^ signalling reverts to store-independent activation.

### 3.4 Nephrin rescue in ND ciPod modifies FFA signalling to that of WT ciPod in normal and minimal Ca^2+^ conditions

**Fig. 4:**
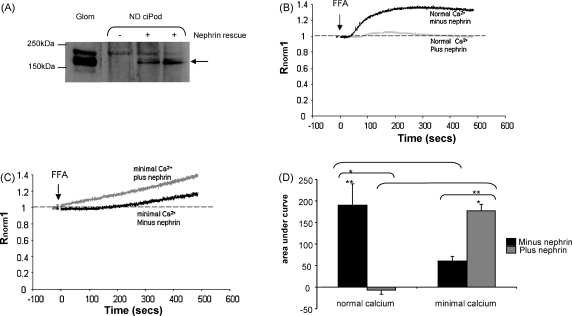
Nephrin rescue in ND ciPod modifies FFA signalling to that of WT ciPod in normal and minimal Ca^2+^ conditions. Proliferating ND ciPods are readily transfectable compared to differentiated ND ciPods and were therefore used to rescue nephrin expression in these cells. (A) Proliferating ND ciPods were transfected with plasmid alone or with nephrin. Cell and glomerular (glom) lysates were western blotted for nephrin. Proliferating ND ciPods, transfected with nephrin or empty plasmid were loaded with Fura-2AM. (B) Representative traces of the effect of FFA in ND ciPods with and without nephrin rescue. (C) Representative traces of the effect of FFA in minimal Ca^2+^ on ND ciPod with and without nephrin rescue Experiments from (B) and (C) are summarised in (D), *n* = 5, 4, 5 and 5 respectively. One way ANOVA *p* < 0.0001, Bonferroni post hoc tests indicated. *p < 0.05, **p < 0.01, ***p < 0.001.

To confirm that the results seen in ND ciPods in response to FFA were specific to nephrin we carried out nephrin rescue experiments. Transfection of differentiated ciPods is technically difficult, but proliferating ciPods (at the permissive temperature of 33 °C) are readily transfectable using conventional techniques (as described in methods) and show a similar amplitude in increased [Ca^2+^]_i_ in response to FFA as differentiated ciPod (data not shown). The protein expression of nephrin at 180 kDa was confirmed in transfected proliferating ND ciPod by Western blotting, in Fig. 4A.

Proliferating ND ciPods transfected with nephrin or empty plasmid were loaded in Fura-2AM, perfused in buffer containing normal Ca^2+^ and stimulated with FFA. Transfected nephrin inhibited the [Ca^2+^]_i_ response to FFA in ND ciPods (AUC_500_; −7.1 ± 9.5 s compared to 190.1 ± 50 s, *p* < 0.05, Fig. 4B and D), demonstrating that the pattern of activation by FFA is nephrin-dependent in ciPods. Having established this nephrin rescue model we went on to confirm that nephrin is involved in store-dependent FFA activation. Proliferating ND ciPods were perfused in buffer containing minimal Ca^2+^ then stimulated with FFA. In cells transfected with empty vector the absence of external Ca^2+^ blocked the response to FFA, as seen in differentiated ND ciPods. The transfection of nephrin led to a partial recovery of independence on external Ca^2+^ and to store-dependence (AUC_500_ without nephrin: 60.7 ± 10 s compared to with nephrin; 177.4 ± 15, *p* < 0.01 paired *t*-test, Fig. 4C and D). This confirms that functional nephrin expression negatively regulates FFA induced signalling, as has been reported for TRPC6 in transfected HEK293 [32], and is at least partially responsible for the store-dependent activation by FFA in WT ciPods.

### 3.5 Nephrin modifies the Ca^2+^ signal induced by FFA in ciGEnC

**Fig. 5:**
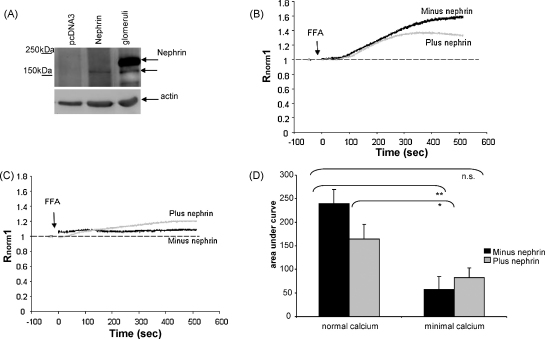
Nephrin modifies the Ca^2+^ signal induced by FFA in ciGEn*C*. ciGEnC were successfully transfected with wild type nephrin (*n* = 3). (A) Glomeruli were used to indicate the expected MW of nephrin (180 kDa) and actin was used to demonstrate equal loading. GEnC, transfected with nephrin or pcDNA3 were loaded with Fura-2AM. (B) Representative traces of the effect of FFA in GEnC with and without transfected nephrin in normal Ca^2+^. (C) Representative traces of the effect of FFA in minimal Ca^2+^ on GEnC with and without transfected nephrin. Experiments from (B) and (C) are summarised in (D), *n* = 6, 4, 6 and 4 respectively. One way ANOVA *p* < 0.01, Bonferroni post hoc tests indicated. *p < 0.05, **p < 0.01, n.s. = non significant.

To understand whether the [Ca^2+^]_i_ store release induced by FFA stimulation in ciPod is dependent on nephrin specifically or whether it is dependent on other podocyte proteins we carried out similar experiments in GEnC. GEnC transfected with nephrin or empty plasmid were loaded with Fura-2AM, perfused in buffer containing normal extracellular Ca^2+^, then stimulated with FFA. The transfection of GEnC with nephrin was confirmed by Western Blotting (Fig. 5A). The effect of nephrin on the amplitude of the response to FFA was then investigated. In nephrin transfected GEnC, the [Ca^2+^]_i_ response induced by FFA was reduced compared to pcDNA3 (AUC_500_ with nephrin; 164 ± 32 compared to pcDNA3; 240 ± 30 Fig. 5B and D). This response was consistent with that seen for nephrin rescued ND ciPod (Fig. 4B), but to a lesser magnitude, demonstrating the inhibitory effects of nephrin on FFA induced Ca^2+^ activation. To assess the effects of nephrin on the FFA-induced release of Ca^2+^ from intracellular stores, GEnC cells were perfused with buffer containing minimal Ca^2+^ and stimulated with FFA. In pcDNA3 transfected GEnC, minimal extracellular Ca^2+^ reduced the response to FFA as previously demonstrated in untransfected GEnC (Fig. 1A). The presence of nephrin induced an increased Ca^2+^ response to FFA (AUC_500_ plus nephrin; 83 ± 21) which was not significantly different to cell activation by FFA in normal Ca^2+^ either with or without nephrin (One way ANOVA, *p* < 0.01 overall) and, again, was consistent with the nephrin rescue experiments in ND ciPod but to a much lesser degree (Fig. 4C and D). This suggests that nephrin is partially responsible for the release of Ca^2+^ from intracellular stores induced by FFA (seen in ciPod), even when expressed in endothelial cells.

### 3.6 Cell activation by FFA does not affect release from Ca^2+^ stores in GEnC, but induces release from Ca^2+^ stores in ciPod in a nephrin dependent manner

**Fig. 6:**
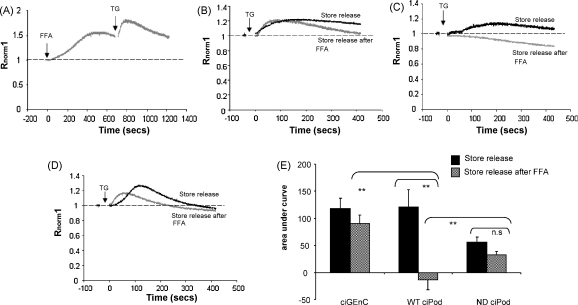
Cell activation by FFA does not affect release from Ca^2+^ stores in GEnC, but induces release from Ca^2+^ stores in ciPod in a nephrin dependent manner. GEnC, WT ciPod and ND ciPod, loaded in Fura-2AM were stimulated first with FFA, then TG. (A) A representative trace of ciGEnC stimulated with FFA followed by TG. (B) Representative traces of TG in ciGEnC, WT ciPod and ND ciPod that were first stimulated with FFA (store release after FFA) as in A. (C) All cell types were stimulated with TG in minimal Ca^2+^ without FFA to assess total store capacity. The amount of store release by TG after FFA treatment was then expressed as a percentage of total store capacity for each cell type, *n* = 5, *n* = 7 and 5 respectively. One way ANOVA *p* < 0.001. Bonferroni post hoc test significances indicated ***p < 0.001, **p < 0.01 n.s. = not significant.

The release of Ca^2+^ from intracellular stores following stimulation of TRPC6 is not well documented, yet the results in this manuscript suggest that it may occur in ciPod. Two potential activation pathways were considered: (1) that FFA was acting to release Ca^2+^ via the generation of secondary messengers, or (2) that FFA was directly acting on the membrane of the [Ca^2+^]_i_ store. To test these hypotheses the effect of cell activation by FFA on store depletion was examined. Cells were loaded in Fura-2AM and perfused with buffer containing normal Ca^2+^. Cells were stimulated with FFA, or vehicle for 8 min or until the response started to diminish, then cells were stimulated with TG to inhibit the endoplasmic reticulum Ca^2+^ ATPase (SERCA), and release Ca^2+^ from intracellular stores, as demonstrated in ciGEnC in Fig. 6A. Store release by TG after FFA stimulation does not result in capacitative Ca^2+^ entry (CCE) because FFA blocks the store-dependent TRPC channels at the plasma membrane [33]. To assess the proportion of store Ca^2+^ released by TG after FFA stimulation, results were compared to cells perfused in minimal Ca^2+^ and stimulated with TG alone (total store Ca^2+^ capacity). In ciGEnC FFA minimally affected store release by TG (69.1 ± 12.5% of total store capacity, Fig. 6B and C). In contrast, in WT ciPod FFA stimulation inhibited further store depletion by TG (−0.13 ± 0.2% of total store capacity, One way ANOVA *p* < 0.001 with Bonferonni post hoc test, *p* < 0.01, Fig. 6B and C). Interestingly, the availability of Ca^2+^ from stores in ND ciPod was smaller compared to the other cell lines, although TG stimulation could still activate capacitative Ca^2+^ entry in the absence of FFA demonstrating that the concentration of TG used did deplete the stores. In ND ciPod FFA stimulation had a small effect on subsequent store depletion by TG (57.5 ± 12.3% of total store capacity, Fig. 6B and C), which was significantly different to ciGEnC. These results suggest that in contrast with ciGEnC, stimulation by FFA in normal podocytes induces the release of Ca^2+^ from stores, and does so at least partially in a nephrin-dependent manner.

### 3.7 Expression and distribution of TRPC6 in ciPod and ciGEnC

**Fig. 7:**
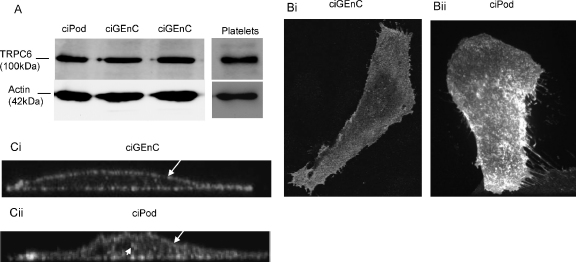
Expression and distribution of TRPC6 in ciGEnC and ciPod compared to a platelet positive control. Lysates from ciPod and cGEnC were Western blotted and probed using anti-TRPC6 and anti-actin antibodies. (A) Representative images of ciGEnC (Bi) and ciPod (Bii) were microinjected with pcDNA3TRPC6 and immunofluorescence was carried out using an anti-TRPC6 antibody. Cells were imaged using confocal microscopy. X–Y stacks of ciGEnC and ciPod in (B) are shown in (Ci) and (Cii) respectively.

Next, we established the expression and distribution of TRPC6 in both ciGEnC and ciPod. Endogenous TRPC6 appeared to be expressed in similar levels in both ciPod and ciGEnC (Fig. 7A). We could not detect endogenous TRPC6 above background by immunofluorescence using the commercially available TRPC6 antibodies; therefore cells were microinjected with WT TRPC6. In ciGEnC, overexpressed TRPC6 was diffusely distributed throughout the plasma membrane (Fig. 7Bi and Ci, arrows), yet in ciPod it appeared to be localised within microsomal-like structures in two populations; within the plasma membrane (arrows) and cytoplasm (arrowhead, Fig. 7Bii and Cii).

### 3.8 Nephrin clustering affects the distribution of TRPC6 in HEK293 cells and potentially FFA induced changes in [Ca^2+^]_i_

**Fig. 8:**
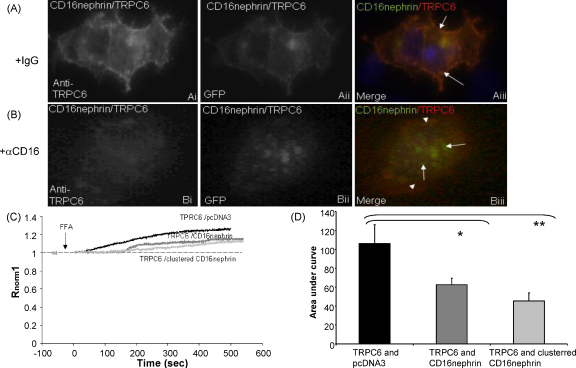
Nephrin clustering affects the distribution of TRPC6 in HEK293 cells and potentially FFA induced changes in [Ca^2+^]_i_. HEK293 were transiently transfected with TRPC6 and CD16-nephrin for 24 h. Cells were incubated with either secondary antibody alone (Ai–iii) or 1 mg/ml anti-CD16, then secondary antibody (Bi–iii) before fixing and immunostaining for TRPC6. Dapi nuclear staining is also shown in the merged images. Transfected HEKs, loaded with Fura-2AM were incubated in 1 mg/ml anti-CD16 for 10 min or vehicle, then incubated with 1 mg/ml mouse IgG for 10 min and finally stimulated with 200 μM FFA. Representative traces are shown for cells stimulated with FFA that were transfected with TRPC6 and pcDNA3 or TRPC6 and CD16-nephrin under control and experimental conditions (C). Data from C are summarised in (D) as area under the curve (*p* < 0.01 One way ANOVA, *n* = 3, 6 and 5 respectively, Bonferroni post hoc tests indicated *p < 0.05, **p < 0.01).

To examine whether nephrin signalling was necessary to affect TRPC6 behaviour, the distribution of TRPC6 was examined after the activation of nephrin through clustering, as described previously [31]. In brief, the inclusion of the CD16 domain on the extracellular side of the nephrin molecule enables a stimulated clustering of the nephrin by treatment with an anti-CD16 antibody, and the inclusion of the GFP tag on the nephrin enables visualisation of the nephrin. HEK293 cells were co-transfected with TRPC6 and CD16-nephrin. Nephrin clustering was induced using mouse anti-human CD16 in conjunction with a secondary anti-mouse AF488 antibody and TRPC6 immunostaining was carried out. There was limited fluorescent signal detected at the appropriate wavelengths in the absence of transfected cDNA. In TRPC6/CD16-nephrin transfected HEK cells incubated with secondary antibody alone TRPC6 and nephrin colocalised predominantly along the plasma membrane (Fig. 8 Ai–iii, yellow). In contrast, when nephrin clustering was induced using anti-CD16 the two populations separated (Fig. 8B i–iii, green). CD16-nephrin was lost from the plasma membrane, and TRPC6 showed a distinctly reduced linear plasma membrane distribution, with increased punctate staining throughout the cell. These results suggest that upon clustering nephrin dissociates from TRPC6 and both proteins relocalise.

To examine whether clustering of CD16-nephrin had an effect on FFA induced Ca^2+^ activation, similar experiments were carried out on cells loaded with Fura-2AM and changes in [Ca^2+^]_i_ were measured. Incubation with anti-CD16 followed by mouse IgG had no effect on R_norm_, neither did incubation with mouse IgG alone (data not shown). In TRPC6/pcDNA3 transfected HEK293 cells, FFA stimulated an increase in [Ca^2+^]_i_ (AUC_500_ 106 ± 20) which was significantly reduced in untreated TRPC6/CD16-nephrin transfected cells (AUC_500_ 62 ± 5, *p* < 0.05 post hoc test) and reduced further in anti-CD16 treated (nephrin clustered) TRPC6/CD16-nephrin transfected cells (AUC_500_ 46 ± 8, one way ANOVA, *p* < 0.01, *p* < 0.05 post hoc test, Fig. 8B and C). This reconfirms that the presence of intracellular nephrin negatively regulates [Ca^2+^]_i_ activation by FFA and that nephrin clustering may have an additional effect on FFA Ca^2+^ activation.

## 4 Discussion

We have shown that, in contrast to GEnC, store-depletion in ciPod inhibits FFA-induced Ca^2+^ release, conversely FFA stimulation results in the release of Ca^2+^ from stores. Both of these characteristics are lost in the absence of nephrin, which is presumably why they are not observed in ciGEnC. TRPC6 expression in ciGEnC is restricted to the plasma membrane and is diffuse whereas in ciPod it is distributed throughout the plasma membrane and cytoplasm in punctate vesicular-like bodies and FFA-induced Ca^2+^ release is reduced when TRPC6 is redistributed by CD16-nephrin clustering. It is therefore clear from our findings that the FFA-induced changes in [Ca^2+^]_i_, presumably through TRPC6 activation, is markedly different in the two cell types forming the glomerular filtration barrier.

In the absence of significant extracellular Ca^2+^, FFA curiously induced a marked increase in overall [Ca^2+^]_i_ in ciPod (Fig. 2C). It is known that reduced extracellular Ca^2+^ from 2 to 0.05 mM causes an increase in whole cell current through TRPC6 due to a relieved block by Ca^2+^, whereas an increase in extracellular Ca^2+^ from 2 to 100 mM attenuates this [4]. However, when the authors measured whole cell current and changes in [Ca^2+^]_i_ simultaneously in TRPC6 transfected HEK293 cells in 20 μM extracellular Ca^2+^, OAG stimulation only affected the TRPC6 current, and did not stimulate Ca^2+^ entry. This suggests that even though the current through TRPC6 had increased, there was no effect on Ca^2+^ because the extracellular source was too low and there was no secondary source. In ciPod however FFA stimulation, which increases whole cell current through TRPC6 in low extracellular Ca^2+^, *did* appear to result in an increase in [Ca^2+^]_i_ due to a secondary source other than extracellular Ca^2+^.

The FFA induced [Ca^2+^]_i_ responses do appear inconsistent in their activation pattern between cell types and conditions. This is probably due to the native channel configurations upon which FFA is acting i.e. native TRPC6 heterotetramers may well differ between GEnC and ciPod therefore altering its activation kinetics [34]. Since there is little known about native channel activation in these cell types it would be hard to predict the activation patterns.

At closer inspection of the literature, the FFA-induced release of Ca^2+^ from intracellular stores that we describe in ciPod may have already been demonstrated serendipitously in another cell type; work carried out on neurons in the late 1990s by Lee et al. [35] demonstrated an FFA-dependent increase in [Ca^2+^]_i_ through a non-selective cation channel, which was eliminated by pre-treatment with TG. When this work was carried out it was not known that FFA activated TRPC6. Interestingly, TRPC6 is highly expressed in neurons [36] and podocytes share many characteristics with them [37]. In addition, it was recently shown that native TRPC6 could play a role in CCE in platelets in association with the ER Ca^2+^ sensor, STIM1 in combination with the plasma membrane Ca^2+^ channel Orai 1, but acted as a SOC in association with TRPC3 [34], indicating that the location of TRPC6 mediated Ca^2+^ entry is plastic.

In recent years a similar mechanism of Ca^2+^ release from stores induced by FFA as the one we describe in ciPod has been described for TRPM8, a temperature sensitive branch of the TRP family. Tsuzuki et al show that the menthol-induced increase in [Ca^2+^]_i_ through TRPM8 in dorsal root ganglion cells was eliminated by TG pre-treatment. The authors also demonstrate that menthol directly induced the release of Ca^2+^ from TG sensitive stores through TRPM8 [38]. Also, TRPM8 was suggested to act as a “Ca^2+^ release channel” on stores in prostate carcinoma cell lines [39]. Our data suggest that TRPC6 is organised in vesicular-like bodies or microsomes in ciPod, supporting functional evidence that FFA induces release of Ca^2+^ from stores such as the endoplasmic reticulum or Ca^2+^ containing microsomes.

Although we and many other groups have described an effect of FFA on TRPC6 Ca^2+^ signalling, the use of FFA to activate TRPC6 coems with a number of assumptions. Recent work carried out by Tu et al [40] suggests that FFA stimulates the release of Ca^2+^ from mitochondria in neuronal and HEK293 cells, which depresses SOC activity. Although there is no evidence that FFA can induce the release of Ca^2+^ from mitochondria in podocytes, we cannot rule it out entirely. However, it would have to occur in ciPod alone and not ciGEnC or HEK293 cells since FFA had no effect when HEK293 (including TRPC6 over expressing HEK293 [25]) and ciGEnC (Fig. 1A) were incubated in minimal extracellular Ca^2+^, suggesting that there was no mitochondrial release. In our experiments we demonstrated that depletion of TG sensitive stores inhibited subsequent FFA-induced Ca^2+^ entry, whereas Tu et al. show that the effects of FFA were independent of TG-sensitive stores which led them to investigate mitochondrial Ca^2+^ release. Therefore, whether FFA stimulates TRPC6 activation directly or indirectly it certainly stimulates a different Ca^2+^ activation pathway in ciPod from GEnC, and does so only in the presence of the podocyte-specific protein nephrin.

It appears that nephrin negatively regulates the amplitude of cell signalling by FFA in ciPod and is at least partially responsible for the FFA-induced depletion of stores. Interestingly, nephrin also appears to reduce the amplitude of cell signalling by FFA in GEnC. There is also a suggestion that it may be involved in GEnC store-release, however these results are not very dramatic, which suggests that nephrin is only partially responsible for the FFA-induced store-release in ciPod and that other podocyte factors are likely to play a role. This area will need further research to elucidate the other factors involved. We demonstrated that TRPC6 and CD16-nephrin colocalised at cell-cell junctions in an artificial expression system, suggesting that TRPC6 interacts with the cytoplasmic tail of nephrin. Phosphorylation of the cytoplasmic tail of CD16-nephrin by clustering (as described before [31]) induced the dispersion of TRPC6 from cell-cell junctions into vesicular-like bodies, yet CD16-nephrin and TRPC6 no longer colocalised. The presence of CD16-nephrin with TRPC6 significantly reduced FFA-induced Ca^2+^ activation and nephrin clustering reduced the Ca^2+^ activation by FFA further still. Therefore, it appears that nephrin activation may enhance its negative regulatory effects on FFA-induced Ca^2+^ signalling and its effects on TRPC6 distribution may also be responsible for communication of TRPC6 with internal Ca^2+^ stores.

The results from this manuscript suggest that FFA activates a Ca^2+^ channel that is functionally different in human podocytes than in human glomerular endothelial cells. We have previously shown that FFA can activate TRPC6 in ciPod [25] and have good supporting evidence that TRPC6 localisation is different between ciPod and GEnC and that clustering of nephrin changes the distribution of TRPC6 leading to a reduction in Ca^2+^ entry by FFA stimulation. Although not definitive, together these results strongly suggest that FFA stimulates TRPC6 activation in both ciPod and GEnC and the cell differences seen are due to differences in TRPC6 activation.

Patients described by Winn et al. [20] and Reiser et al. [21] with TRPC6 mutations have all developed FSGS, which is thought to initiate with podocyte injury [41]. Podocytes have become the focus of attention in recent years because of other gene mutations that have been identified which encode proteins at, or in association with the podocyte slit diaphragm and cause hereditary nephrotic syndrome, such as NPHS1 (nephrin) [22], ACTN4 (α actinin-4) [42] and NPHS2 (podocin) [43], of which TRPC6 is the latest addition. Thus it may not be that surprising that, of the cell types that form the filtration barrier, TRPC6 is uniquely activated in podocytes and might therefore be more sensitive to deleterious affects of mutations. This work highlights an interesting hypothesis to explain why TRPC6 mutations only seem to induce glomerular disease, despite physiological involvement in many different cellular systems.

In conclusion, we have demonstrated that FFA stimulates a different Ca^2+^ activation pattern in ciPod, which suggests that TRPC6 may be functionally different between human glomerular endothelial cells and podocytes through nephrin regulation, which in light of recent findings may play a role in the progression of glomerulosclerosis such as in FSGS.
